# The State of Squamate Genomics: Past, Present, and Future of Genome Research in the Most Speciose Terrestrial Vertebrate Order

**DOI:** 10.3390/genes14071387

**Published:** 2023-07-01

**Authors:** Simone M. Gable, Jasmine M. Mendez, Nicholas A. Bushroe, Adam Wilson, Michael I. Byars, Marc Tollis

**Affiliations:** School of Informatics, Computing, and Cyber Systems, Northern Arizona University, Flagstaff, AZ 86011, USA; smg655@nau.edu (S.M.G.);

**Keywords:** squamates, genome sequencing, genome assembly, phylogenomics, transposable elements

## Abstract

Squamates include more than 11,000 extant species of lizards, snakes, and amphisbaenians, and display a dazzling diversity of phenotypes across their over 200-million-year evolutionary history on Earth. Here, we introduce and define squamates (Order Squamata) and review the history and promise of genomic investigations into the patterns and processes governing squamate evolution, given recent technological advances in DNA sequencing, genome assembly, and evolutionary analysis. We survey the most recently available whole genome assemblies for squamates, including the taxonomic distribution of available squamate genomes, and assess their quality metrics and usefulness for research. We then focus on disagreements in squamate phylogenetic inference, how methods of high-throughput phylogenomics affect these inferences, and demonstrate the promise of whole genomes to settle or sustain persistent phylogenetic arguments for squamates. We review the role transposable elements play in vertebrate evolution, methods of transposable element annotation and analysis, and further demonstrate that through the understanding of the diversity, abundance, and activity of transposable elements in squamate genomes, squamates can be an ideal model for the evolution of genome size and structure in vertebrates. We discuss how squamate genomes can contribute to other areas of biological research such as venom systems, studies of phenotypic evolution, and sex determination. Because they represent more than 30% of the living species of amniote, squamates deserve a genome consortium on par with recent efforts for other amniotes (i.e., mammals and birds) that aim to sequence most of the extant families in a clade.

## 1. Introduction

Squamates (Order Squamata) are a near-globally distributed clade of reptiles including ~11,000 extant species of lizards, snakes, and amphisbaenians [1]. The large number of species and extensive phenotypic variation observed across squamates make them one of the most diverse and successful of the vertebrate orders. However, as next-generation sequencing technologies have enabled access to vast genomic datasets for model and nonmodel organisms, squamates have been relatively underrepresented in genomic datasets compared to other groups, such as mammals and birds [2,3], until recently. This has placed limitations on our knowledge of the genomic mechanisms underlying phenotypic traits among squamates, the branching order of squamate diversification, and the origins and extent of genomic variation across different groups of vertebrates. Here, we review major milestones in squamate genome sequencing, describe limitations and challenges to obtaining genomic data for the group, and discuss promising areas of research using squamate genomes that can shed light on universal mechanisms in biology. We highlight the need for orienting future squamate genome sequencing efforts toward targeting a more complete taxonomic sampling across the order with higher quality assemblies.

## 2. What Are Squamates (and “Reptiles” for That Matter)?

**Figure 1:**
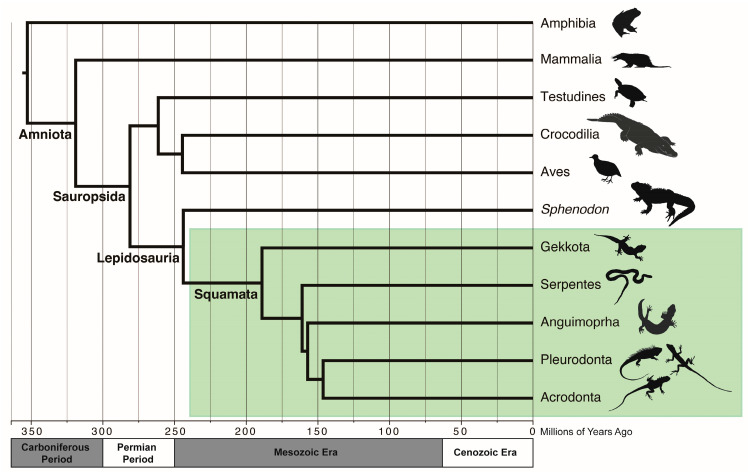
Phylogenetic relationships and evolutionary history of amniotes. Relationships and approximate divergence times for the major extant amniote lineages. Amphibians (Amphibia) are the outgroup. Amniotes arose ~310 million years ago (MYA) in the Carboniferous Period, and after the branching off of synapsids (leading to modern mammals), sauropsids began radiating in the Permian Period. The Lepidosauria emerged ~250 MYA. The only extant nonsquamate lepidosaurian is the tuatara (*S. punctatus*). Most major groups of Squamata (green box, represented here by Gekkota, Serpentes, Anguimorpha and the pleurodont and acrodont iguanians) diverged during the Mesozoic Era. Divergence time estimates were taken from www.timetree.org [20]. Geological timescale is approximate. Animal silhouettes from www.phylopic.org under the public domain.

Squamates and humans share a common history with birds, mammals, and other reptiles, which together form a unique lineage on the Tree of Life called amniotes. Amniotes (Amniota) first appeared during the Carboniferous Period ~318 million years ago (MYA) [4]. The watertight amniotic egg was a key innovation that allowed amniotes to diversify and thrive on land, and by ~300 MYA amniotes had split into two lineages: sauropsids (Sauropsida; including living reptiles, birds, and their extinct relatives) and synapsids (Synapsida; including mammals and their extinct relatives) (Figure 1). Mammals (Class Mammalia) are the only extant synapsids with around 6400 living species [5], including the egg-laying monotremes (e.g., platypuses and echidnas), the pouched marsupials, and placental mammals (eutherians, including humans). Other synapsid lineages, such as pelycosaurs and therapsids, were diverse and abundant during the Permian and Early Triassic Periods, but were later replaced by sauropsids in the Mesozoic Era (also known as the “Age of Reptiles”) [4].

Living sauropsids include all reptiles and birds and are classified into two main lineages [6]. The first is a turtle and archosaur clade (Archelosauria [7]). While the relationship between turtles and other amniotes was highly debated during the 20th century, genomic studies confirm a turtle/archosaur sister relationship [7,8,9,10]. Archosaurs include the descendants of the most recent common ancestor of all living crocodilians and birds. All archosaur descendants had split into separate crocodilian and avian lines by the Triassic Period ~220 MYA, and were supremely successful during the Mesozoic Era [4]. Living crocodilians (Order Crocodylia) are the only remaining crocodilian-line archosaurs. Avian-line archosaurs include pterosaurs and all dinosaurs, most of which disappeared from the fossil record after the end-Cretaceous mass extinction. The ~11,000 extant species of birds (Class Aves) are the only surviving dinosaurs.

The sister taxon to the archelosaurs are the lepidosaurs, which share several synapomorphies including overlapping scales (Lepidosauria; Greek for “scaled lizards”). Within Lepidosauria there are two extant taxonomic orders: the once-diverse Rhynchocephalia (with the tuatara, *Sphenodon punctatus*, as the single living species) and Squamata. Squamates include all extant lizards, snakes, and amphisbaenians (i.e., worm lizards), with a fossil record extending to the Early Triassic ~240 MYA [11]. Squamates are by far the most speciose nonavian reptile clade [1], are found on every continent today except Antarctica, and feature diverse adaptations involving limblessness, venom systems, parity, carnivory, herbivory, and marine and aquatic lifestyles. The diversity and familiarity of squamates have enthralled humans for centuries, taking the cultural roles of both evil mediums [12,13,14] or venerated deities [15,16] in folklores around the world. Squamates also serve as integral study organisms for research in physiology [17], pharmaceutical therapies [18], evolution [11], and animal behavior [19].

## 3. The Era of Amniote Genomes and The Long Road to Squamate Genome Representation

Vertebrate comparative genomics started with a purposeful orientation toward humans and other mammals. While complete genome sequences were already available for model eukaryotes with smaller genomes (i.e., *Saccharomyces*, *Arabidopsis*, *Caenorhabditis elegans*, *Drosophila*) [21,22,23,24], the completion of the Human Genome Project [25,26] set off a race to further determine the origins of functional elements in the human genome, particularly genomic regions involved in human diseases. This is because purifying selection on functional elements should result in conservation at the sequence level [27], which would be made more apparent through comparisons of organisms that share a relatively recent common ancestor with humans.

**Figure 2:**
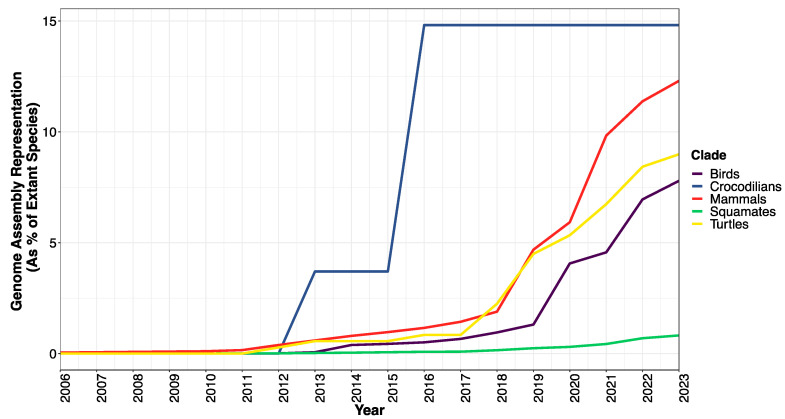
Genome database representation for amniotes, including squamates, during the 21st century. The proportion of extant species of squamate with a complete genome assembly available on NCBI as of spring 2023 is far below that for birds and turtles, and, to a greater degree, crocodilians and mammals.

The mouse (*Mus musculus*) and rat (*Rattus norvegicus*) genomes were made available in 2002 and 2004, respectively [28,29], and the sequencing of the chimpanzee (*Pan troglodytes*) genome in 2005 and rhesus macaque (*Macaca mulatta*) genome in 2007 filled some of the gaps in mammalian and primate evolution [30,31]. The Broad Institute went on to sequence and release 29 mammalian genomes in 2012 [32], and, fueled by group efforts such as Genome10K [33] and the Earth BioGenome Project [34], the Zoonomia consortium recently published 200 genomes representing 56% of mammalian families [3,35]. Other endeavors such as the DNA Zoo (DNAzoo.org) and the Vertebrate Genomes Project (www.vertebrategenomesproject.org) have released their own mammalian genome assemblies (Figure 2).

A few years, after the publication of the human genome, draft genomes for birds started to appear on GenBank (Figure 2). The draft genome of a chicken (*Gallus gallus*) was released in 2004 to fill the evolutionary gap between mammals and other model organisms [36]. The next avian genome to be fully sequenced and published was the songbird model zebra finch (*Taeniopygia guttata*) in 2010 [37]. The Avian Phylogenomics Consortium published 48 avian genome assemblies after their “phase I” in 2014, followed by a 2020 “phase II” analysis of 363 genomes covering 92% of bird families [2,38].

The first available reptile genome sequence was that of a squamate, the green anole lizard (*Anolis carolinensis*), published in 2011 [39]; however, additional squamate genomes were released at a relatively slower rate compared to other amniotes. The first two published snake genomes were that of the Burmese python (*Python bivattatus*) and king cobra (*Ophiophagus hannah*) in 2013 [40,41]. After 2019, high-quality assemblies existed for the Komodo dragon [42], a few geckos [43,44,45], several species of lateratans, including a tegu [46] and multiple lacertids [47,48], and several advanced snakes. As of early June 2023 there were 90 genome assemblies for squamates in GenBank, representing 0.82% of squamate species and 34.7% of squamate families (last accessed 31 May 2023) (Supplementary Materials).

Despite the progress in reptile genomics, a few key squamate lineages were still lacking high-quality genome sequences in early 2023, including members of the dibamids, scolecophidians (or “nonadvanced” snakes), amphisbaenians, and most acrodont iguanian groups. Lack of sampling from these lineages introduces many phylogenetic gaps that encompass important and ancient events in squamate evolution, such as at the root of the squamate phylogeny ≤ 240 MYA [11] and key events in the Earth’s history, such as the breakup of Pangea. Several major events in squamate evolution happened in relatively quick succession, such as the evolution of venom glands [49] and the advanced snake radiation [50]. These events make useful calibration points for hypotheses about genome evolution at deep timescales; however, the long branch lengths associated with sampling gaps preclude the ability to capture patterns of genomic variation before and after these events. While whole genome sequences for warm-blooded amniotes are close to a complete representation of their respective diversity, at least at the family level, a genomic Age of Reptiles has yet to truly begin.

### 3.1. Sequencing and Assembly Quality Impact the Utility of Genomes

**Figure 3:**
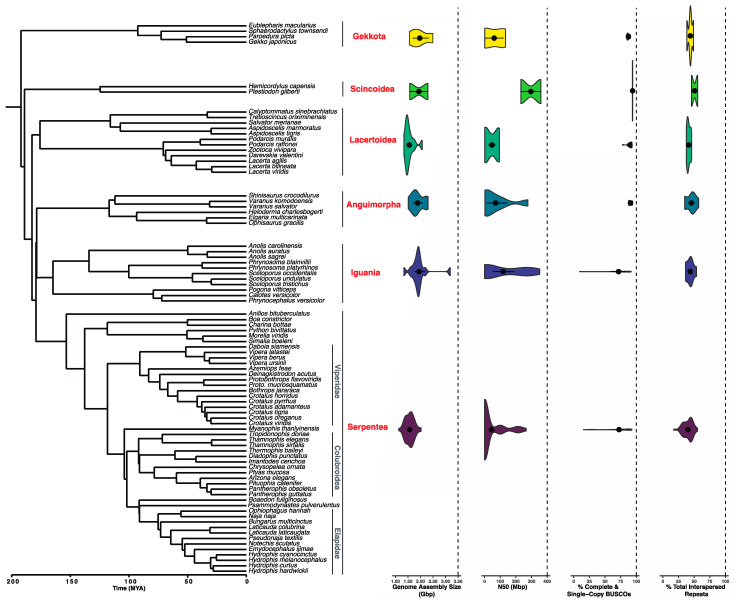
Comparison of genome assembly size, scaffold N50, percentage of complete and single-copy BUSCO genes, and percentage of total interspersed repeats across major Squamata clades with colors organized by clade, based on 83 publicly available genome assemblies. Assembly size and scaffold N50 are reported from NCBI metadata. Total interspersed repeat content is from de novo RepeatMasker output. Mean squamate assembly size is 1,696,615,317 bp (1.70 Gbp) with a standard deviation of 322,492,546 bp. MYA = millions of years ago.

The green anole genome put forth by the Broad Institute was one of the last of the “old generation” 7X Sanger-sequenced projects [39]. Next-generation sequencing (NGS, or second-generation sequencing) has significantly lower costs per megabase through its massively parallel sequencing design, and many available squamate genomes have been sequenced using Illumina platforms. While NGS provides genome coverage (i.e., 100X), which is a necessary step for a quality assembly, shorter NGS reads of 100–150 bp have difficulty spanning many types of interspersed repeats. This presents a problem for squamate genomes in particular, since they are rich in repetitive elements such as nonlong terminal repeat (non-LTR) retrotransposons [51,52]. Due to their naturally high repetitive content, squamate genomes assembled using only Illumina reads tend to be highly fragmented (Figure 3 and Supplementary Materials).

Long-read sequencing platforms (or third generation) from Pacific Biosciences (PacBio, Menlo Park, CA, USA) and Oxford Nanopore Technologies (ONP, Oxford, United Kingdom) have significantly decreased in cost [53]. With reads in the tens of thousands of bases in length, these technologies are especially useful in spanning repetitive regions and lead to better estimates of interspersed repeat content in genomes [54,55]. Because long reads have higher error rates than NGS, sequencing the same genome with both short- and long-read technologies is becoming the gold standard [56], with several recent squamate genomes assembled using this hybrid approach [57,58].

All sequenced genomes must be assembled to align and merge reads into a full genome representation. Usually, short or long reads are assembled into stretches of contiguous sequence called contigs, which are then properly ordered and oriented during a process called scaffolding. Building genome scaffolds starts by sequencing read pairs which span longer insert sizes than the typical NGS library. Scaffolding libraries can include Illumina mate-pair libraries that span a few thousand base pairs (mate pair library sizes of 3 kb, 10 kb, and 25 kb are common), while other methods like Hi-C often result in insert sizes of hundreds of thousands of base pairs [59]. Hi-C libraries that are sequenced with Illumina short read pairs can effectively join contigs to form fully or near-chromosome-length scaffolded genome assemblies for many nonmodel organisms [60]. An additional step may include optical mapping (i.e., BioNano, San Diego, CA, USA), which can effectively map scaffolds to physical chromosomes [61].

To ensure high assembly quality, important decisions must be made early on in the project, in particular what coverage or “depth” of sequencing will be obtained. Coverage is an estimate of the number of times each site in the genome is represented by a sequenced read. Higher coverage improves the chances that sequencing errors will be corrected. Postassembly, genomes are evaluated mainly using three metrics: contiguity, completeness, and correctness. Contiguity is quantified using the N50 statistic, which is similar to a weighted median length. Starting with the largest contig (or scaffold), the contig lengths are added together until the cumulative sum is half of the total assembly length, and the N50 is the length of shortest contig in that list. Long read assemblies result in much higher contig N50s, making them useful for capturing structural variation and longer genic regions, which are typically fragmented in Illumina-based assemblies.

Completeness can be assessed with BUSCO (Benchmarking Universal Single-Copy Orthologs), a tool that measures the amount of clade-specific conserved orthologs in an assembly compared to the expected amount for the taxon of interest [62]. If a high proportion of complete and single-copy BUSCOs are found in an assembly relative to what is expected, then the assembly is likely to contain a large degree of quality genic information. The third metric is more difficult to quantify: correctness refers to the accurate order and location of contigs reflecting that of the true genome. The amount of misjoins, translocations, and the number of duplicate BUSCOs have been used to describe correctness [63].

### 3.2. Where Does the Availability and Quality of Squamate Genomes Currently Stand?

At the beginning of 2023 there were 83 squamate species with available genomes on NCBI, representing ~0.75% of living species (Figure 3), and by June of that year, the number increased to over 90. The taxonomic distribution and quality of publicly available squamate genomes as of this writing (last accessed 31 May 2023; collected from NCBI and other databases, Supplementary Materials) ranges drastically for each major squamate clade (Figure 3). Several squamate families were completely absent from the whole genome databases (i.e., all of Dibamidae, Amphisbaenidae and several families of Scolecophidia). There is a relative overabundance of genomes available for some snake families, such as Viperidae, for which scaffold N50 and BUSCO completeness vary widely (Figure 3). The same applies to Elapidae and Colubroidea, likely reflecting interest by researchers studying venom evolution [41,64,65]. By early June 2023, an amphisbaenid genome became available on NCBI via the Vertebrate Genomes Project, reflecting a rapidly changing field of squamate genomics.

**Figure 4:**
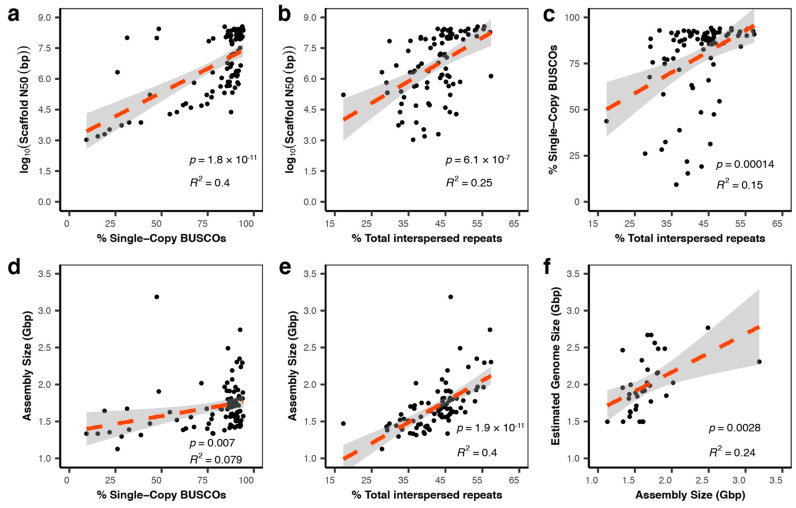
Relationships between genome assembly metrics across 91 squamate genomes. We analyzed contiguity (N50), assembly size (bp), gene completeness (% BUSCOs), and repetitiveness (% total interspersed repeats). (**a**) Assembly contiguity predicts gene completeness. (**b**) Assembly contiguity predicts interspersed repeat content. (**c**) Genome assemblies with high predicted gene content contain a similarly greater interspersed repeat content. (**d**) Larger genomes do not contain a larger percentage of complete genes. (**e**) Larger genomes contain more interspersed repeats. (**f**) A positive significant relationship between estimated genome size from the Animal Genome Database and assembly size.

We analyzed the relationships between assembly metrics of 91 publicly available squamate genomes in terms of their assembly size in bp, scaffold N50, and the proportion of single-copy BUSCOs found in the genome (accessed 15 April 2023; Figure 4 and Supplementary Materials). For the squamate genomes, N50 was highly predictive of gene content as measured by the proportion of found single-copy BUSCOs (*p* = 1.8× 10^−11^; R^2^ = 0.4). N50 was also predictive of interspersed repeat content (*p* = 06.1 × 10^−7^; R^2^ = 0.25). In the meantime, predicted gene content predicted interspersed repeat content but without a large effect (*p* = 0.00014; R^2^ = 0.15). We also found that while assembly size did not have a strong effect on predicted gene content (*p* = 0.007; R^2^ = 0.079), assembly size did predict interspersed repeat content (*p* = 1.9 × 10^−11^; R^2^ = 0.4), highlighting the fact that repetitive DNA, and not the number of protein-coding genes, plays an important role in determining genome size [66]. It is worth noting that measured genome size from the Animal Genome Size Database [67] predicted assembly size for squamates, although experimental error in genome size estimation or the inability of some sequencing projects to capture the entire genome likely reduces the effect size of this relationship (R^2^ = 0.24). Our analysis suggests that N50, particularly for contigs, should be a prioritized metric in order to maximize detection of both coding and noncoding regions of the squamate genome.

When planning a squamate genome project and depending on its goals, it may be best to initially focus on quality contig assembly from long reads for initial analysis to maximize genic coverage followed by the possibility of acquiring scaffolding libraries (i.e., Hi-C) that increase contiguity as the model system develops and new questions arise. For instance, the genome of the brown anole lizard (*Anolis sagrei*) has been improved to the chromosome level [68] in order to support its development as the first reptile to be successfully used in CRISPR-Cas9 genome editing experiments [69] (see below).

The next sections will highlight areas of research that stand to benefit the most from squamate whole genome data. The first is phylogenomics. We will review the major disagreements in deciphering the squamate phylogeny, methods of high-throughput phylogenomics and their effects on the inference of squamate relationships, and the promise whole genomes carry to settle or sustain persistent phylogenetic arguments in squamates. Then we will explain how squamate genomes provide opportunities to understand the evolution of genome size and structure in vertebrates via transposable elements. We will cover methods of transposable element annotation and analysis and how squamates in many ways make ideal models for understanding transposable element evolution. We will also discuss how squamate genomes can contribute to venomics research, studies of phenotypic evolution (including CRISPR and evo–devo studies), and sex determination research.

## 4. Putting the “Genomics” in Squamate Phylogenomics

Phylogenies can be used to visualize evolutionary relationships, understand trait evolution, and form the foundation of many predictive models in biomedical research for drug development, forensics, and gene function [70,71]. Phylogenetic methods rely on inferred homology of characters and the distributions of character states across species in order to make inferences on relationships between organisms [72]. The advancement of NGS technologies has enabled phylogenetics to evolve into a newer field, often referred to as “phylogenomics”. Several comparative genomic studies of vertebrates have shed light on the relationships among the major branches of amniotes, such as the placement of turtles in the amniote phylogeny [8,9,10,73], and to determine that DNA substitution rates are correlated with phenotypic or species diversity among reptiles [74,75], although this is a topic still under debate [76]. Here, we will discuss how complete and accurate genomes will be extremely useful for the testing of several hypotheses about squamate phylogenetic relationships.

Genome-wide studies of squamate relationships have the potential to resolve longstanding debates about squamate diversity. The earliest cladistic analyses of squamates used morphological, fossil, ecological, and behavioral data, supporting two main squamate lineages [77]: Iguania, which comprises ~2000 living lizard species as diverse as iguanas, anoles, and chameleons; and Scleroglossa, within which all limbless and limb-reduced groups formed a subclade consisting of the dibamids, amphisbaenians, and all snakes (i.e., Serpentes). In contrast, the first phylogenetic studies of squamates to use molecular data disagreed with the Scleroglossa–Iguania dichotomy [78], and led to an alternative hypothesis of squamate evolution. In most of these studies, either Dibamia or Gekkota were placed at the root of the squamate phylogeny, followed by Scincoidea (i.e., Scinciformata), Lacertoidea (i.e., Laterata), and a clade that included Anguimorpha (varanids, Gila monsters, and glass lizards), Iguania, and Serpentes [79,80]. This disagreement between morphologists and molecular biologists studying squamates led to a classic schism in systematics [81].

A strong argument for the molecular hypothesis of squamate evolution was the discovery of Toxicofera, or the “venom clade” [49]. Several snake species from previously classified nonvenomous clades were found to actually harbor venom proteins, which originated from venom genes that are shared not only among snakes but also iguanians and anguimorphs [49]. This required a revision of the widely accepted theory that venom had multiple independent origins across squamates. Instead, venom was an ancestral state followed by subsequent losses (such as in the nonvenomous iguanians). Toxicofera unites three major squamate groups—about 60% of living squamate species—and agrees with the consensus of molecular phylogenies of squamates: there is no molecular support for Iguania as the sister taxon to all other squamates nor for a Scleroglossa clade [82].

Genome-scale datasets that have been used to reconstruct the squamate tree of life include anchored hybrid enrichment loci [82], protein-coding genes [83], and ultraconserved elements [84], often containing hundreds of markers and tens of thousands of aligned base pairs. However, while some of these studies boast very high statistical support for their resulting topologies, areas of phylogenetic uncertainty remain, including the relationships between the major Toxicoferan groups and the placement of either Dibamia or Gekkota at the root of the squamate tree. This pattern of generally high certainty within but disagreement between studies suggests that while a large number of loci lower the overall sampling variance in support of a split in the tree, it will not reduce gene tree–species tree discordance, which is when the topologies of individual gene trees conflict with the underlying species tree [85,86].

Phylogenomic discordance can be driven by biological factors, such as incomplete lineage sorting and introgression (i.e., “true” discordance), and also artifactual factors such as model violations and issues with data quality. Multilocus phylogenetic methods such as “supergene” concatenation most often assume that all sampled loci can be explained by the same underlying tree [87], and so are not equipped to account for discordance. Many “species tree” approaches are consistent with the multispecies coalescent and can account for discordance from incomplete lineage sorting. Species tree methods typically entail summing over a large number of individual gene trees followed by their incorporation into a species tree [87].

Interestingly, squamates may present a “perfect storm” of problems commonly found to drive discordance in molecular phylogenetic datasets. For instance, the phylogenetic signal for older divergences (such as the Triassic or Jurassic-aged divergences of most squamate suborders) may be reduced by genetic saturation, where multiple substitutions at the same site accumulate over time, which most substitution models for phylogenetic analysis are not equipped to handle [88,89]. Also, the failure of molecular phylogenetics to recover Scleroglossa suggests that limblessness and limb reduction are convergent traits that evolved numerous times across the history of squamates. Convergence has had profound problems for many criteria-based methods in phylogenetics that rely on maximum parsimony, such as those in most morphological analyses [90]. In addition, rapid evolutionary radiations, such as many splits within squamates, are often associated with short internodes and long descendant branches, which create an “anomaly zone” where gene trees inconsistent with the species phylogeny are more probable [91,92].

Despite a large number of markers, phylogenomic methods used for squamates still have largely relied on reduced representations of the genome, and are less likely to incorporate differential evolutionary patterns and rates such as across coding and noncoding regions. The future of squamate phylogenomics, therefore, lies in the ability to sample the entire genome, encompassing the widest possible range of coalescent histories and substitution rates, and the ability to parse the signal from the noise in phylogenomic reconstruction. These methods have already been applied to other amniote groups. For instance, an analysis of millions of orthologous parsimony-informative sites across annotated features of the mammalian genome showed that coding regions were predominant drivers of discordance while noncoding regions contained far more data that agreed with the species tree [93]. A recent phylogenomic analysis based on the complete genomes of turtles revealed strong support for the species tree across coding and noncoding regions, yet a considerable amount of discordance that could be explained by genetic saturation at more ancient branches and incomplete lineage sorting and/or introgression at more recent branches [8]. These studies benefit from a large number of potential sites or loci that allow strict filtering without loss of signal.

To demonstrate the utility of complete genome assemblies in reconstructing the squamate tree of life, we downloaded 91 squamate genome assemblies—plus human, chicken, alligator, and the tuatara as outgroups. We then assessed each genome for the presence of 7453 single-copy orthologous protein sequences from the sauropsid orthoDB database with BUSCO v5 and computed multiple sequence alignments for each ortholog with MAFFT [94]. We relied on protein sequences for this analysis to minimize the effects of saturation, which is less prominent in protein compared to nucleotide sequences. We retained only alignments ≥100 amino acids in length, >75% taxa representation, and with fewer than 15% gaps using AMAS [95], resulting in 6050 genes for downstream analysis. We computed gene trees for each accepted alignment in IQ-TREE2 [96] using model testing, and used the resulting genealogies in a species tree analysis with ASTRAL-III [97], assessing branch support with local posterior probabilities [98]. Gene concordance factor is a measure of the proportion of gene trees supporting a split in the species tree, while site concordance factor represents the proportion of variable sites in an alignment supporting the split [86]. To measure gene tree–species tree discordance, we computed gene and site concordance factors for each branch in the squamate species tree with IQ-TREE2.

**Figure 5:**
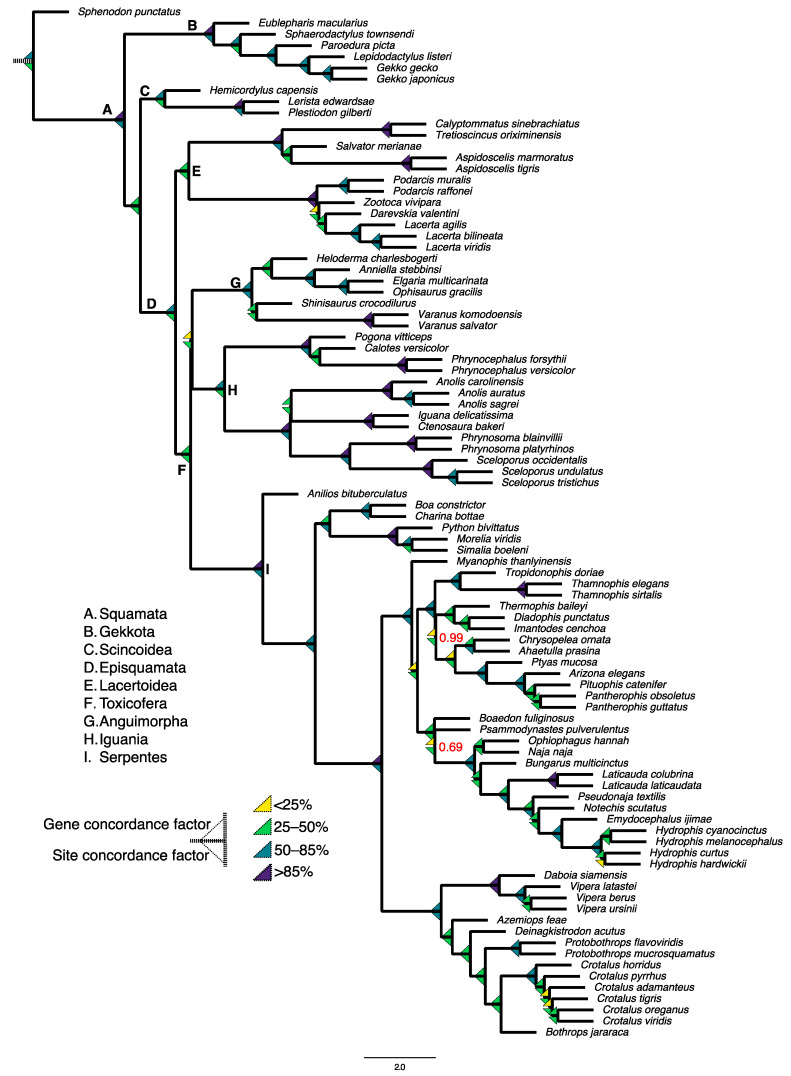
Species tree reconstruction and discordance analysis of 91 squamate species based on 6050 protein sequences extracted from complete genome assemblies. We downloaded publicly available genome assemblies for 91 squamates, and extracted orthologous protein sequences and aligned and filtered sequences according to Gable et al. (2022) [8]. Gene trees were inferred with IQ-TREE2 using model testing, and a species was constructed given the gene trees using ASTRAL-III [97]. All branches received 100% posterior support except where indicated. Gene and site concordance factors were computed in IQ-TREE2 [86].

We obtained complete local posterior support for all splits in the squamate species tree (Figure 5), except for internal branches within colubrid (0.99 posterior support) and elapid (0.69 posterior support) snakes. The overall topology was consistent with most molecular phylogenetic studies of squamates. However, concordance factors were highly variable across the squamate phylogeny, in particular at key branches including the branch leading to lateratans (Lacertoidea), as well as the ancestral branch of the three toxicoferan clades (Iguania, Anguimporha, and Serpentes) and the branch uniting the snake families Colubridae and Elapidae.

Our analysis of a large number of protein sequences reveals that there is much disagreement among gene genealogies for squamates. By employing these genome-wide approaches, squamate comparative genomics may yield resolutions to these longstanding conflicts, as well as causal explanations for the underlying disagreements about the topology of the squamate tree of life. For instance, machine learning techniques can help decipher between biological and artefactual discordance [99], and for squamates in particular, artificial neural networks have been used to determine the properties of individual genes that disagree with the species tree [82]. Also, the inclusion of fossil lineages in total evidence based analyses results in more robust estimates of divergence times and evolutionary rates in squamates [76,100]. With the sequencing of additional genomes that can help fill the phylogenetic gaps in squamate evolutionary history, squamates will continue to be a dynamic model for studying how genome-wide heterogeneity affects phylogenetic results.

## 5. Transposable Elements in Squamates and Other Amniotes

What applies to squamate species and phenotypic diversity equally applies to their genomic diversity [101]. Across vertebrates, only a small fraction of the genome comprises protein-coding genes, while the rest is made up of vast regions of repetitive noncoding DNA such as transposable elements (TEs). Found in all eukaryote genomes, TEs are parasitic DNA sequences that move about the genome via a process known as transposition [102] and are major drivers of genome size, accounting for ≥60% of the human genome [103]. Studies of squamate TEs have revealed a complex level of biological organization with its own set of dynamics and distinct evolutionary history [39,51,52,101,104,105]. Understanding these TE evolutionary dynamics in squamates will shed light on fundamental processes governing genome size, structure, and function.

TEs are divided into two main classes based on their mechanism of transposition. Class I elements are the retrotransposons, which use a copy-and-paste method via an RNA intermediate that is reverse transcribed into a new DNA locus. Retrotransposons are further classified into those containing “long terminal repeats” (LTRs) and those lacking repetitive flanks (non-LTRs), and are the dominant elements across most eukaryotic genomes [106]. Within the non-LTR retrotransposons are the fully autonomous Long Interspersed Nuclear Elements (LINEs), such as LINE-1 found in human and most other amniotes, which encode an endonuclease and reverse transcriptase required to generate and insert a copies of themselves into the host genome. Most of these LINE inserts are inactivated through truncation during reverse transcription [107]. The other group of non-LTR retrotransposons are the nonautonomous Short Interspersed Nuclear Elements (SINEs), which rely on LINEs for their replicative machinery.

Class II elements are the DNA transposons. The “cut-and-paste” transposons include a transposase that removes the transposon and inserts its sequence at a different genomic location. DNA transposons are particularly abundant in nonavian reptilian genomes [101], with 23 superfamilies each with numerous subfamilies [108]. Helitron elements likely mobilize via rolling-circle replication [109] and the mechanism of Mavericks/Polintons is still unknown.

### Methods for Analysis of Transposable Elements

Determining the diversity and abundance of TEs in the genomes of squamates and other vertebrates requires bioinformatics tools capable of detecting and analyzing TEs in sequence data. RepeatMasker is a popular software that can use TE consensus sequences as queries in BLAST searches against the genome, outputting annotated lists of repetitive DNA [110]. TE consensus sequences are often calculated using majority-rule based on DNA alignments of individual insertions from specific TE families or subfamilies, and can be found in databases such as RepBase [108] and Dfam [111]. Repeat databases are dominated by model organisms like *Drosophila*, human, chicken, and house mouse, leading to ascertainment biases that underestimate species-specific or clade-specific TEs in nonmodel organisms [112].

One approach to repeat findings in nonmodel organisms is to model repeat consensus sequences de novo with RepeatModeler [113], which uses the repetitive structure of TE copies, identifies conserved regions of verified TE classes, and builds a library of genome- and species-specific consensus repeats. Many genome assembly algorithms erroneously collapse repetitive regions into contigs [60], and some de novo repeat-finding methods model TE consensus sequences based on repetitive kmers found in sequence reads rather than relying on potentially error-prone genome assembly as a first step [114]. Once repeat consensus sequences are obtained from a genome via any of these de novo methods, RepeatMasker can then use them in BLAST queries of RepBase or Dfam to classify and annotate TEs.

The initial analysis of the green anole genome revealed a repeat-rich squamate genome with a high diversity of Class I and II TEs, many of which are recently active and producing copies [39,51,104,105]. One analysis showed that total TE content varied considerably more than expected across snakes, yet TE families were present at similar proportions [40]. Across squamate genomes, total interspersed repeat content, which is driven by TEs, ranges from 25% to >50% of the genome (Figure 3 [52]).

The range in TE abundance across squamates is in stark contrast to relative uniformity in terms of repeat content within mammals and birds, respectively [52,115]. For instance, across most avian genomes studied only ≤10% of genomic content has been attributed to TEs, mostly truncated CR1 retrotransposons [115,116]. One interesting exception to this is woodpeckers and their relatives, which experienced a significant lineage-specific CR1 amplification to comprise ~20% of the genome [117]. Mammalian genomes are TE-rich yet tend to be dominated by LINE and SINE retrotransposons, such as LINE-1 and Alu elements that alone contribute to >30% of the human genome [25], with the exception of vesper bats, which experienced a notable expansion of DNA transposons [118,119].

To visualize the diversity in TE content across squamate genomes, we downloaded the assemblies from 91 squamate species representing 24 families (Supplementary Data) and modeled repeat family consensus sequences de novo for each assembly using RepeatModeler2.0, followed by repeat family consensus sequence classification for each genome with RepeatMasker v4.1. For each genome, we then used the classified repeat family consensus sequences in a second RepeatMasker analysis in order to determine the distribution of the relative abundances (in terms of the proportion of the total genome) of LINEs, SINEs, LTR retrotransposons, and DNA transposons across each analyzed squamate family.

**Figure 6:**
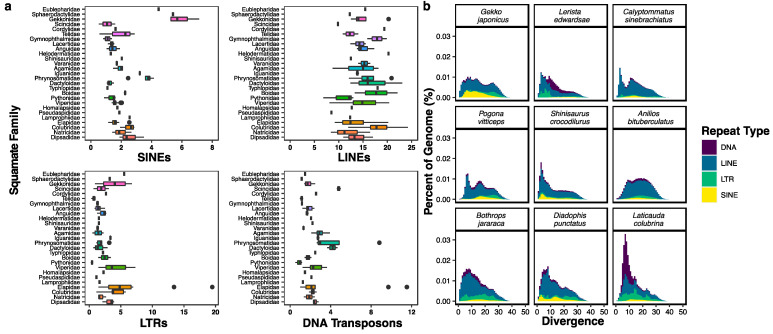
(**a**) Abundance of the four main TE subclasses across 25 squamate families based on 91 genomes/species. Families are ordered by taxonomic relationship. Vertical bars represent median, black circles represent outliers. (**b**) Repeat landscapes for nine squamate genomes representing key lineages. Divergence is in terms of Kimura 2-parameter distance from family consensus.

We found considerable variation in TE content across squamate genome assemblies, mirroring previous results based on short read datasets [52]. For instance, we observed a relative overabundance of SINEs in the genomes of all geckos analyzed (six species; families Gekkonidae, Sphaerodactylidae, and Eublepharidae) as well as in the five species of phrynosomatid (Figure 6a). The abundance of LINEs also differs greatly among squamate genomes; in particular we found a range of ~7–~25% of genomic proportions attributed to LINEs within colubroid snakes alone. We also observed relative overabundances of both LTR retrotransposons and DNA transposons in elapid snakes, while LTR retrotransposons were nearly absent in the python genome.

The long-term patterns of TE accumulation and loss in squamate genomes have been measured in numerous ways, including comparisons of TE abundance and diversity between genomes above the species level [52,75,104,105], and population genetic-level analysis of TE insertion polymorphisms that measure the relative roles of drift and selection in determining the fate of TEs in a genome [120,121]. Most of these methods rely on pairwise sequence divergence between each TE insertion and its family consensus sequence, which is used as a proxy of element age [122]. Specifically, divergence is calculated as the percentage of base substitutions relative to the consensus sequence, with low divergence implying a younger TE and high divergence pointing to an older TE [123]. A histogram plotting the proportion of the genome composed of each TE family according to different levels of divergence is referred to as a repeat landscape, and the repeat landscapes of multiple genomes can be compared to shed light on differing dynamics of TE activity across species [124].

The differences in TE abundance and diversity between squamate genomes may be driven by stark lineage-specific differences in TE activity. To understand the differences in TE activity between major groups of squamates, we generated repeat landscapes for representatives of six major squamate clades (Gekkota, Lacertoidea, Anguimorpha, Iguania, Serpentes; Figure 6b). First, we ran RepeatMasker on each genome using the species-specific de novo consensus library with the -a flag to generate alignments of each TE insertion to its family consensus sequence, and the calcDivergence.pl script, which estimates the Kimura 2-parameter (K2P) sequence divergence of each TE insertion to its family consensus sequence. We then plotted histograms of the proportion of each genome consisting of each TE type (LINE, SINE, LTR retrotransposon, DNA transposon) in bins of 1% divergence.

We found evidence of several lineage-specific recent TE expansions across the representative squamates. For instance, a large proportion of DNA transposons ≤15% divergence in the genome of the elapid snake *Laticauda colubrina* is consistent with a relative overabundance of DNA transposons that appear to be unique to this group of snakes [125] and, therefore, may be the result of a relatively recent burst of DNA transposon activity. Meanwhile, there is a spike of recent LINE activity at ≤10% in the gymnopthalmid *Calyptommatus sinebrachiatus*, as well as recent activity of all TE types in *Shinisaurus crocodilurus*, *Diadophis punctatus*, and *Daboia siamensis*. Our analysis of squamate repeat landscapes reveals that many squamate genomes are highly transpositionally active.

The movement of TEs in squamate genomes can have profound effects on genome structure and function. While most TE-induced mutations are deleterious or nearly neutral [126], some TE activity can lead to beneficial outcomes for the host, including TE domestication [127], exaptation by existing protein-coding genes, insertion and subsequent impact on regulatory regions [128], and TE-mediated increased genome plasticity [129]. Squamates have an overabundance of TEs at loci within the organized *HOX* gene clusters, which help govern body axis orientation during development. These TEs may cause expression changes in the *Hox13* and *Hox10* genes controlling the expansion of the caudal and thoracic skeletal regions in the corn snake *Pantherophis guttatus* [130]. In addition, the accumulation of TEs in *HOX* regions were associated with speciation rate estimates as well as changes in morphological traits known to be important in the adaptive radiations of *Anolis* lizards [131].

Novel TEs can be horizontally transferred between unrelated species, where a lack of TE-specific silencing mechanisms by the host leads to bursts of novel TE activity. Galbraith et al. (2021) identified a lineage-specific autonomous DNA transposon from the *Harbinger* family in the snake genus *Laticauda*, a group of highly venomous sea krait species [125]. The *Harbinger–Snek* transposon was horizontally transferred, likely from a sea urchin genome, which allowed the transposon to proliferate through the krait genome unchecked given their new host lacked any defense mechanisms to halt TE activity. Insertion time estimates places the insertion event just prior to the divergence of the *Laticauda* crown group, and analysis of *Harbinger–Snek* insertion locations suggests that this TE likely played a role in the genus’s adaptation to an amphibious marine environment through altered gene expression.

The abovementioned study also identified a key difference in TE dynamics at the species level, finding evidence for the accordion model between the two *Laticauda* genomes. In this model, the mechanism of DNA loss of recently expanded TEs is explained by nonallelic homologous recombination events (NAHR, also called unequal crossing over), where sequences with high similarity from different locations in the genome recombine [132]. NAHR is one of the most common mechanisms leading to deletions and rearrangements, and high-copy TE families are an ideal substrate to promote such events. In the *Laticauda* kraits, evidence suggests that the *L. laticaudata* genome underwent a series of NAHR deletions following mass transposon expansion, allowing the species to retain a genome size similar to related terrestrial species [125].

Large-scale differences in TE dynamics have been shown between major vertebrate clades as well; a positive correlation between DNA gain and DNA loss was found in birds, but not in mammals, suggesting that the avian genome structure is governed by deletions more so than mammals [115]. Regarding dynamics and size impact, some researchers support the theory that genome size is an adaptive trait influenced by selective pressures, while others argue that genome size is simply the result of neutral evolution, as pointed to by an “accordion model” of genome size evolution [66]. Although genome size varies more than 60,000 fold among eukaryotes [133], genome size is tightly conserved across squamates [67]. Previous estimates found an average ~0.2-fold size variation in genome assembly length for squamates [52]; with our expanded taxon sampling we find even less variation with a ~0.14-fold difference in assembly length across squamates (Figure 2). The fact that squamate genomes show significantly more variation in TE content and activity than bird genomes [115] suggests that lineage-specific TE-host dynamics in squamates help to constrain genome size in the face of TE activity.

Genome assembly quality can greatly affect the estimation of genomic repeats. As previously mentioned, short reads sequencing can make accurate characterization of TE content difficult due to the short read lengths and highly repetitive makeup of TEs, particularly when the focal element is longer than the read length (e.g., LINEs). Long-read sequencing technology not only improves the quality of assemblies but also allows reads to span full repetitive regions and increase TE annotation accuracy. Even in gold standard reference assemblies of model organisms, long reads increased TE insert detection by 26–57% and identified hundreds of new TE variants associated with adaptive evolution [134]. These methods should be applied to the repeat-rich genomes of squamates in order to fully capture order-wide genomic variation.

## 6. Genomic and Phenotypic Evolution in Squamates

Linking genomic and phenotypic evolution has expanded from early efforts of simple genotype–phenotype mapping to the use of genome-wide loci and comparative genomics [135]. Many traits vary across squamates, showing rapid turnover, such as parity modes [136] and asexual reproduction (i.e., parthenogenesis [137,138]), while others are unique lineage-specific features like the ballistic tongue of chameleons [139]. To understand the genotype–phenotype–fitness relationship, particularly for complex, polygenic traits, requires integrating genomic and phenotypic data, collecting in situ population-level experiments, and quantifying fitness effects [140]. These resource-heavy studies have been limited to reduced representation genomic data [141] or to a handful of species with available genome and transcriptome data [17]. Validating functional effects of identified mutations is logistically easier in a laboratory setting, which explains why many available squamate genomes are docile species common in the pet trade (e.g., *Pogona vitticeps*, *Eublepharis macularius*, *Gekko gecko*, *Salvator merianae*, *Shinisaurus crocodilurus*) [142]. However, artificial selection and laboratory experiments do not account for the role of demographic and selective impacts of evolution in natural populations, which may influence genomic architecture (see [143]).

Using whole genome assemblies, researchers can identify both the specific genes controlling traits of interest as well as their position, contextualizing the genomic architecture of trait evolution as was recently performed for pit viper chemoreception [144]. Outside of coding regions, conserved noncoding regulatory elements are a main contributor to phenotypic diversity across the animal kingdom through controlling gene expression [145]. A recent study examining regulatory elements controlling limblessness in squamates found that the convergence of this trait likely resulted from lineage-specific changes in similar molecular pathways [146]. Most associations between conserved noncoding elements and impacted phenotypes are unknown (especially in squamates), but convergent evolution of regulatory elements is linked to loss of flight in paleognath birds, and “reverse genomics” methods show great promise in mapping genome-wide regulatory elements to a large set of phenotypes [147,148].

The latest advances in genomics, such as CRISPR, have now developed genome-wide screening methods to target thousands of genes in a single injection [149]. Phenotypic analyses in squamates using any CRISPR/Cas9-mediated knockout methods are difficult due to unique reproductive characteristics in squamates such as pliable eggshell and a lack of airspace. However, two recent studies successfully produced the first genetically modified reptiles using an anole (*Anolis sagrei*) and gecko species (*Paroedura picta*) [69,150], opening the door to a new frontier of gene function testing and evolutionary-development model systems.

## 7. The Contribution of Squamate Genomics to Other Diverse Fields of Research

### 7.1. Venomics

Venom delivery systems are a complex phenotypic trait present in anguimorph lizards in the genera *Heloderma* (beaded lizards, Gila monster) [151] and *Varanus* (although this is still debated; see [152,153]), as well as the medically important Viperidae, Elapidae, and Colubroidea snake clades [154]. Venom is composed of varying amounts of peptides, larger proteins, and other organic molecules, mainly employed for predation or defense [155,156]. Venom systems provide a unique model for studying predator–prey coevolution, complex genotype–phenotype mapping, and evolution of multigene families [157,158]. Because of the high target specificity unique to venom toxins, venoms are also a promising source of novel compounds for new drug therapies and biotechnological innovations [159]. The highly cited success of the blood pressure drug captopril developed from *Bothrops jararaca* venom peptides in 1981 triggered a wave of interest in venom-derived pharmaceuticals [160], including the popular obesity and type 2 diabetes drugs, semaglutide (e.g., Wegovy, Ozempic), modeled after a hunger-regulating hormone in *Heloderma suspectum* venom [161].

The king cobra (*Ophiophagus hannah*) was the first venomous snake to have its genome sequenced, revealing and providing genome-wide support for the importance of gene duplication of housekeeping genes followed by recruitment into snake toxin-producing functions (i.e., co-option of venom genes) [41]. However, the lack of comparative whole genome analyses across venomous and nonvenomous taxa leaves the underlying genomic mechanisms of these recruitment events unknown. Whole genome studies may complement transcriptome and proteome data for venomous species, improving accuracy of functional annotation and allowing for gene variant identification. Noncoding DNA harbors information on cis-regulatory functions implicated in controlling venom composition [158,162]. In addition to duplication–recruitment events, gene loss has also been found to impact venom evolution, driven by TE invasion [163]. Identification of the underlying genomic basis and mechanisms producing unique peptide and compound structures will support biomedical research in novel treatments. Unraveling the evolutionary history of squamate venom will require comparative genomic approaches with dense sampling across venomous and nonvenomous species.

### 7.2. Sex Determination

The developmental and evolutionary basis for various forms of sex determination across vertebrates is an active area of research, with direct implications to issues of climate change and conservation [164]. Squamate diversity possesses the three main forms of sex determination systems: genotypic sex determination with female heterogamety (ZZ/ZW; [165,166]), male heterogamety (XX/XY; [167]), and temperature dependence [168]). Some species have mixed systems with both genotypic and environmental sex determination, such as the snow skink (*Niveoscincus ocellatus*), which transitions to a temperature-dependent system in certain climates [169]. Multiple evolutionary transitions between the three forms occurred during squamate evolution [170]; however, the mechanisms controlling sex chromosome evolution, turnover, and gene dosage remain largely unknown due to a lack of high-quality assemblies and annotations of squamate autosomes and sex chromosomes.

Recently, a reannotation of the *Sphaerodactylus townsendi* (Puerto Rican Sandy Geckolet) genome enabled the identification of the candidate primary sex-determining gene that has independently evolved in two fish clades [44]. Reference genomes submitted to public databases (e.g., NCBI) typically do not include either sex chromosome because haplotype-resolved genomes require long sequence reads with low error rates. Furthermore, many species with unique patterns of sex determination lack genomic representation, including scindoidean lizards and many gekkotan families. Squamates are an ideal model system for sex chromosome research, with inter- and intraspecific variation and a complex history of sex chromosome evolution. Due to the diversity and high amount of transitions between sex determination systems in squamate evolution, generalized conclusions based on a chosen reference species is not possible. High-quality (ideally haplotype-resolved) assemblies and annotations as well as broader taxonomic sampling are required to understand this complex life history trait in squamates and amniotes at large.

## 8. Conclusions

Squamates are an important taxon representing a large proportion of extant amniote diversity, and recent advances in DNA sequencing technologies have enabled access to complete genome information for the group that was not possible at the onset of the era of the human genome. We have found that despite a growing number of complete genomes for squamates in the public databases, there is not only considerable variety in genome assembly quality but also gaps in the taxonomic distribution of available genomes for squamates. We suggest that effort should be placed toward obtaining high-quality contigs for key lineages of squamates such as dibamids and amphisbaenians. The fact that an amphisbaenid genome became available at the time of this writing reflects a rapidly changing squamate genomics field which portends many promising future discoveries.

Targeted genome projects for squamates will help establish ancestral states in genome evolution and allow greater accuracy in the reconstruction of squamate evolutionary history. In addition, we assert that squamates deserve a genome consortium on par with Zoonomia for mammals [3,35] and the Avian Phylogenomics Consortium for birds [2,38], in order to coordinate the genome sequencing, assembly, and whole genome alignment for a large number of species representing a majority of squamate families. Harnessing the rich evolutionary history and phenotypic diversity of squamates will continue to shed light on the genomic mechanisms underlying processes of interest to several important subfields of biomedical research, as well as to uncover patterns of diversification for a group with a ~240-million-year fossil history. As ~20% of extant squamate species are threatened with extinction according to recent estimates [171], the time is right for a targeted sequencing initiative that would benefit the conservation of Earth’s most speciose terrestrial vertebrate order.

## Data Availability

The data presented in this study, including genome assembly metadata and accession numbers, gene alignments, trees, and annotations are available at https://doi.org/10.5281/zenodo.7992533 (accessed on 1 June 2023).

## Supplementary Materials

The following supporting information can be downloaded at: https://www.mdpi.com/article/10.3390/genes14071387/s1, Supplementary Data (genome accession numbers used in this study). Supplementary Data S1. Genomes, accession numbers, and metadata including assembly metrics for the genome assemblies used in this study.
